# The Mysterious Case of an Athletic Woman with Recurrent Syncope and a “Normal” Heart

**DOI:** 10.19102/icrm.2019.100701

**Published:** 2019-07-15

**Authors:** Samuel L. Johnston, Mahi Ashwath, Barry London, Jennifer Torgerson, Aleksandra Tosic, Brian Olshansky

**Affiliations:** ^1^Department of Cardiology, University of Iowa, Iowa City, IA, USA; ^2^Department of Internal Medicine, University of Iowa Health Care, Iowa City, IA; ^3^Covenant Cardiology Group, Waterloo, IA, USA; ^4^Department of Cardiology, Mercy Medical Center, Mason City, IA, USA

**Keywords:** Implantable cardioverter-defibrillator, syncope, wearable cardioverter-defibrillator

## Abstract

A 53-year-old female with a history of sports participation presented to a community hospital emergency department for collapse. She was given a LifeVest^®^ wearable cardioverter-defibrillator (WCD) (Zoll Medical Corp., Chelmsford, MA, USA) and scheduled to undergo cardiac magnetic resonance imaging (MRI) with gadolinium enhancement at a tertiary center. However, before the scheduled MRI scan could be performed, she developed tachycardia, for which the WCD alarmed. A dual-chamber implantable cardioverter-defibrillator was subsequently implanted. Assessment of a patient with syncope requires consideration of the idea that a life-threatening and recurrent arrhythmia may be a cause for the problem. However, current guidelines do not cover the routine use of WCDs in syncope. Additionally, the patient described here did not clearly meet United States Food and Drug Administration indications for the provision of an external defibrillator. We present this case to provoke discussion among colleagues regarding this patient’s treatment plan.

## Case presentation

A 53-year-old female, who was a prior collegiate basketball player, presented to a community hospital’s emergency department for syncope. During modestly intense kayaking with her daughter, her arms had suddenly felt numb and, after leaning forward and taking a few deep breaths, she fell out of the kayak and was pulled ashore by her daughter, who described her mother as having fluctuating consciousness over the ensuing half-hour. Two weeks before, she had had a sensation of dizziness prior to experiencing a total loss of consciousness while standing. She awoke shortly thereafter feeling well. She had no history of syncope and no significant family history. Her physical examination was unremarkable.

**Figure 1:**
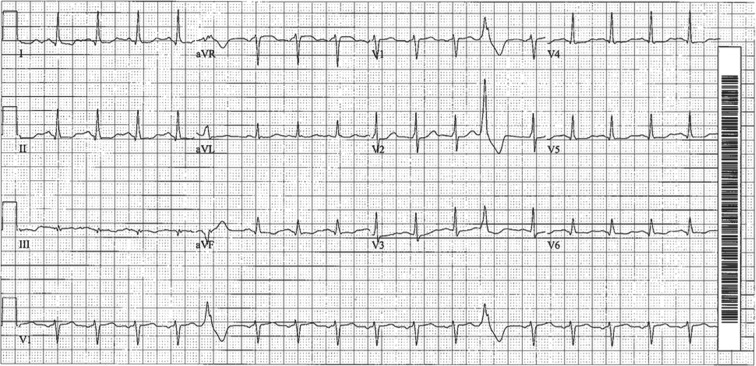
Presenting electrocardiogram.

In the emergency department, her serum potassium was 3.3 mEq/L and troponin was 0.045 ng/mL, and an electrocardiogram showed sinus tachycardia with a corrected QT (QTc) interval of 520 ms and monomorphic premature ventricular complexes (PVCs) (right bundle, superior axis) **(Figure 1)**. The QTc interval normalized with potassium repletion, but the patient continued to experience early- and late-coupled PVCs and rare monomorphic ventricular triplets. Echocardiography and cardiac catheterization findings were normal. A treadmill test showed multifocal PVCs; the QT interval was shortened with exertion. She was discharged on nadolol 60 mg daily, given a LifeVest^®^ wearable cardioverter-defibrillator (WCD) (Zoll Medical Corp., Chelmsford, MA, USA), and scheduled to undergo a cardiac magnetic resonance imaging (MRI) scan with gadolinium enhancement at a tertiary center.

**Figure 2:**
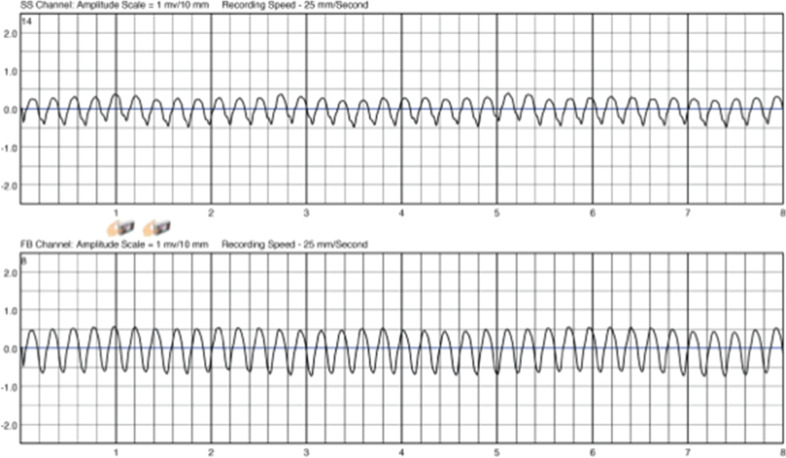
LifeVest^®^ (Zoll Medical Corp., Chelmsford, MA, USA) initiation of tachycardia.

**Figure 3:**
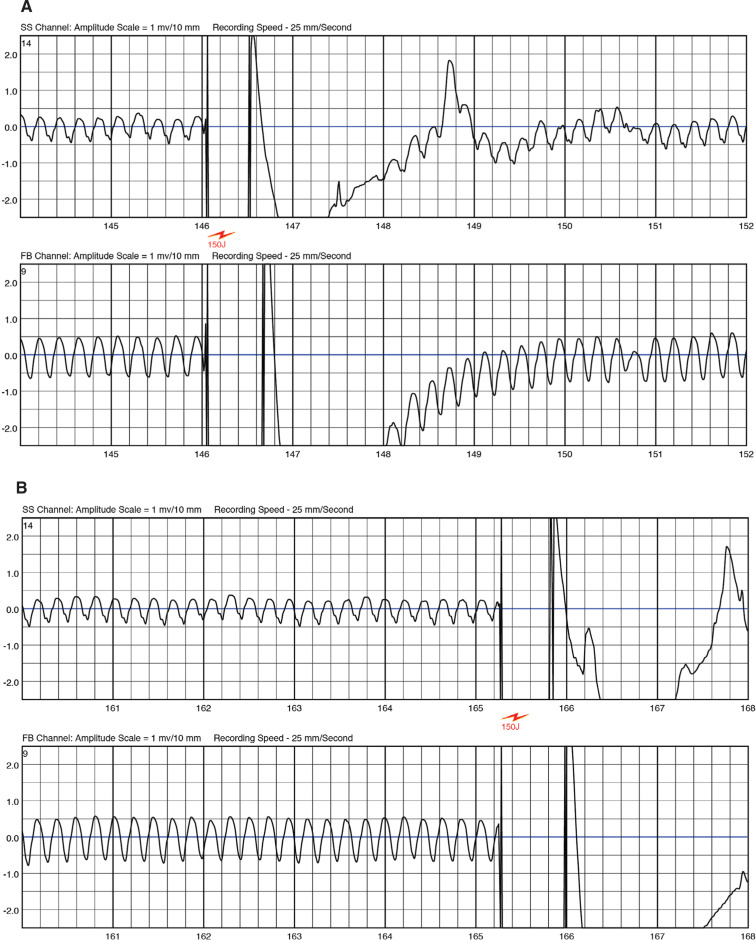

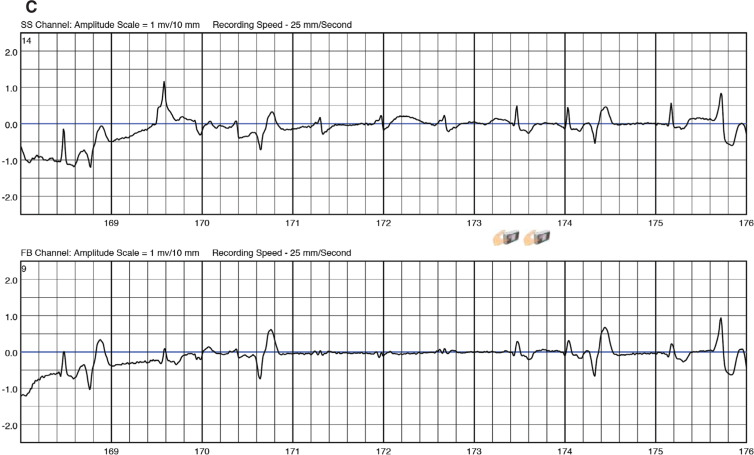
LifeVest^®^ WCD therapies: **A:** Failed shock. **B:** Second shock. **C:** Type 2 break of ventricular tachycardia.

However, before the scheduled MRI scan could be performed, she developed a tachycardia **(Figure 2)**, for which the WCD alarmed. Despite the fast rate (nearly 300 bpm), she repeatedly pressed the response button to suppress a shock. After more than nine minutes, she stopped pressing the response button but was still awake; at this point, she experienced two shocks, the second of which converted the tachycardia to sinus rhythm **(Figure 3)**.

**Figure 4:**
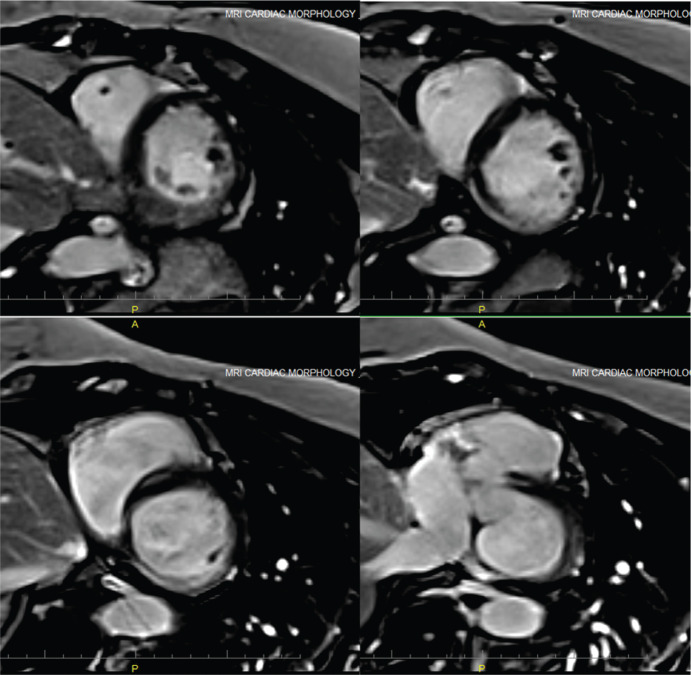
Cardiac MRI scan. Delayed enhancement shows midmyocardial/epicardial scar involving the basal-inferior and inferoseptal walls.

An MRI scan **(Figure 4)** was initiated and demonstrated the presence of delayed myocardial enhancement in the midmyocardium and epicardium, suggesting a nonischemic origin such as myocarditis, amyloidosis, sarcoidosis, or some other form of nonischemic cardiomyopathy.^1^ This patient’s scar was thought to be most consistent with a phenomenon of residual fibrosis from a past viral myocarditis.

**Figure 5:**
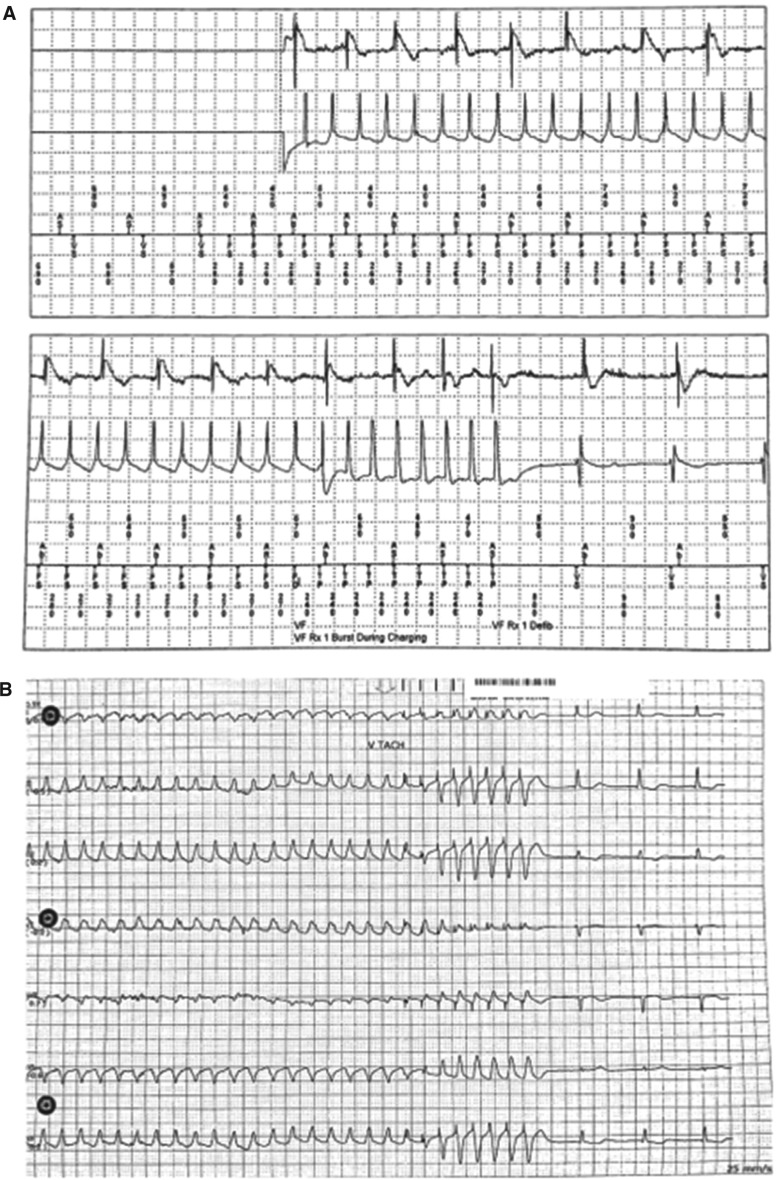
Antitachycardia pacing terminates ventricular tachycardia in conjunction with 80 mg sotalol twice daily (image shows atrioventricular dissociation): **A:** Intracardiac recordings from ICD. **B:** Simultaneous telemetry monitoring.

The location of the patient’s scar suggested a substrate consistent with the morphology of her monomorphic ventricular tachycardia. A dual-chamber implantable cardioverter-defibrillator (ICD) was placed. Based on the fact that she was likely to have a recurrent tachycardia, sotalol 80 mg twice daily was initiated. The next day, she presented a slower but similar ventricular tachycardia that was halted with antitachycardia pacing **(Figure 5)**. The sotalol dose was increased to 120 mg twice daily and she was found to be noninducible via noninvasive programmed stimulation. Subsequently, she has been arrhythmia- and symptom-free.

## Discussion

Assessment of a patient with syncope requires the consideration of the idea that a life-threatening and recurrent arrhythmia may be a cause of the problem. Risk stratification is challenging. This patient’s initial prolonged QTc interval raised the possibility of an inherited channelopathy. However, it was ultimately the patient’s history that most suggested she was at-risk for sudden cardiac death. It is noteworthy that the patient’s first known onset of syncope occurred late in life. More concerning was her fluctuating level of consciousness (likely representing many syncopal episodes) that occurred during her kayak outing. Malignant arrhythmia constitutes a plausible explanation for her symptoms.

Current guidelines^2^ do not address the routine use of WCDs such as the LifeVest^®^ (Zoll Medical Corp., Chelmsford, MA, USA) in patients with syncope. Furthermore, the patient described here did not meet United States Food and Drug Administration indications for external defibrillator use. Indeed, the routine use of a WCD would be inappropriate in most patients presenting with syncope in the absence of structural heart disease. We suggest that a Bayes theorem approach to the risk stratification of patients presenting with syncope be considered (not unlike the risk stratification method employed in assessing patients presenting with the symptom of chest pain).

In light of these considerations, we sought to request input from a panel of experts regarding the following questions:

1. Do you agree with the WCD prescription in this patient? What considerations might help you decide one way or another? Would you have done anything else in addition or anything differently?
2. Why do you think she did not pass out with a rate of 300 bpm?
3. Would you recommend a single-chamber transvenous ICD, dual-chamber transvenous ICD, subcutaneous ICD, or something else for the treatment of this patient?
